# Disentangling the Complexity of the Rumen Microbial Diversity Through Fractionation Using a Sucrose Density Gradient

**DOI:** 10.3389/fmicb.2021.664754

**Published:** 2021-07-08

**Authors:** Ruth Hernández, Hugo Jimenez, Cesar Vargas-Garcia, Alejandro Caro-Quintero, Alejandro Reyes

**Affiliations:** ^1^Computational Biology and Microbial Ecology Group, Max Planck Tandem Group in Computational Biology, Department of Biological Sciences, Universidad de los Andes, Bogotá, Colombia; ^2^Animal Microbiology Laboratory, Agrodiversity Department, Corporación Colombiana de Investigación Agropecuaria – AGROSAVIA, Bogotá, Colombia; ^3^Grupo de Investigación en Sistemas Agropecuarios Sostenibles, Corporación Colombiana de Investigación Agropecuaria – AGROSAVIA, Bogotá, Colombia; ^4^Departamento de Biología, Facultad de Ciencias, Universidad Nacional de Colombia, Bogotá, Colombia; ^5^The Edison Family Center for Genome Sciences and Systems Biology, Washington University School of Medicine, St. Louis, MO, United States

**Keywords:** ruminal microbiota, microbial diversity, sucrose density gradient, fractionation, low abundant microorganisms

## Abstract

The ruminal microbial community is an important element in health, nutrition, livestock productivity, and climate impact. Despite the historic and current efforts to characterize this microbial diversity, many of its members remain unidentified, making it challenging to associate microbial groups with functions. Here we present a low-cost methodology for rumen sample treatment that separates the microbial community based on cell size, allowing for the identification of subtle compositional changes. In brief, the sample is centrifuged through a series of sucrose density gradients, and cells migrate to their corresponding density fraction. From each fraction, DNA is extracted and *16S rRNA* gene amplicons are sequenced. We tested our methodology on four animals under two different conditions, fasting, and post-feeding. Each fraction was examined by confocal microscopy showing that the same sucrose fraction consistently separated similar cell-sized microorganisms independent of the animal or treatment. Microbial composition analysis using metabarcoding showed that our methodology detected low abundance bacterial families and population changes between fasting and post-feeding treatments that could not be observed by bulk DNA analysis. In conclusion, the sucrose-based method is a powerful low-cost approximation to untwine, enrich, and potentially isolate uncharacterized members of the ruminal microbiome.

## Introduction

Ruminants contribute to food security by providing adequate protein and energy to the human population (Matthews et al., 2018). These animals have developed a unique ability to convert plant cell wall carbohydrates into meat and milk, mostly due to the complex and not completely characterized symbiotic microbiota in their rumen (Morgavi et al., 2013; Ribeiro et al., 2016). The ruminal microbiota is composed of diverse populations of obligate anaerobic microorganisms belonging to all life domains such as Bacteria, Eukarya, and Archaea (Nagaraja, 2016), which interact with each other to degrade the plant material and convert it into metabolic by-products and microbial protein (Ribeiro et al., 2016).

For decades, many studies conducted in the rumen have tried to understand the numbers, composition, and function of the ruminal microbial community, mostly using conventional culture-based methods (Morgavi et al., 2013; Ribeiro et al., 2016). These types of methods have only allowed for the isolation and cultivation of about 15% of the estimated bacterial species potentially present in the rumen (Ribeiro et al., 2016). However, for most of the ruminal microorganisms, little is known about their functional role, and many of them have not been taxonomically classified at the genus or species level (Henderson et al., 2015; Puniya et al., 2015).

The modulation of the microbial community structure and function of the rumen has been an area of intense research, particularly in the development of food additives, probiotics, chemical compounds, and diets, which may lead to improved productivity and animal health, as well as the reduction of environmental impacts (Fernando et al., 2010; Poulsen et al., 2013; van Lingen et al., 2017; Teoh et al., 2019; Petri et al., 2020; Stanton et al., 2020). Several recent studies have used the high-throughput sequencing of universal phylogenetic markers such as *16S rRNA* gene to evaluate bacterial community shifts under different treatments (Wang et al., 2017; Yang B. et al., 2018). In many cases, even though these interventions seem to affect animals’ productivity or health, the direct relationship between such treatments and the identification of microbial community members responding to them remains unclear. Various factors might hinder the identification of such differences. For instance, one factor is the population-level diversity not observable with the current methodologies, which obscures the response of specific populations (Caro-Quintero and Ochman, 2015). Another factor is the effect of bulk DNA extraction that destroys the distinct levels of microbial organization and favors the amplification of the most abundant members of the communities, missing important functional organisms that might have lower abundances (Puniya et al., 2015; Eisenstein, 2018). For these reasons, it is necessary to develop new methodological approaches to disentangle the complexity of the ruminal microbiota that favor the recovery of a greater variety of taxonomic groups, allowing a higher resolution for the microbes’ identification affected by treatments and conditions.

The fractionation of the microbial community by cell size and density prior to DNA extraction is an alternative methodological approach that can unravel the enormous microbial diversity present in complex samples. Density gradients are used in different disciplines to separate particles based on size and density (York and Tang, 2015; Lianidou and Hoon, 2018). The principle of the method is simple; first, the sample is deposited on top of either a continuous or step gradient. Then, using centrifugal forces, the different particles in the sample migrate to their corresponding density equilibrium (Brakke, 1951). Different types of media have been used to build density gradients for biological applications; however, one of the most common is a sucrose medium, which has been used for the separation of molecules such as DNA, RNA, and proteins (Raschke et al., 2009; Kleene et al., 2010). In microbiology, the sucrose density gradient has been used for the purification and concentration of viruses (Huhti et al., 2010; Urbas et al., 2011), the separation of *Burkholderia* sp. from a consortium of microorganisms (Chee et al., 2010), and the separation of prokaryotes from eukaryotes (protozoa) in aquatic environments (Garrison and Bochdansky, 2015). Sieving the microbial community through these gradients has the potential for the identification of more subtle differences among less abundant organisms, providing a greater resolution when compared to currently used methods.

In this study, we present a methodology that incorporates a sucrose density gradient as a simple approach for separating ruminal microbial communities by size and density. This approach allows for a more thorough recovery of the diversity of taxonomic groups from the ruminal microbiota compared to commonly used methods for microbial community characterization. To demonstrate the utility of the fractionation methodology, we evaluated the taxonomic diversity shifts of the ruminal microbial community in two different treatments: fasting and post-feeding. We used fractionated and non-fractionated (total) samples and compared the microbial composition using *16S rRNA* gene amplicon sequencing. We hypothesized that density fractionation provides a higher resolution by disentangling the microbial community’s complexity, allowing for a more sensitive identification of taxonomic bacterial groups.

## Materials and Methods

### Experimental Design and Sampling

Three female adults of the creole Colombian cattle breed Blanco Oreginegro (BON) and one male of the Holstein breed were used in the study. These animals were kept at the same location and fed *ad libitum* with fresh grass (*Cenchrus clandestinus*) at the Colombian Agricultural Research Corporation Agrosavia, Tibaitatá (Mosquera, Cundinamarca, Colombia).

Two ruminal samples were collected for each animal after fasting and post-feeding. The animals were initially fed *ad libitum* in a closed grass field, then they were moved to a different enclosure without the availability of food for 16 h. After that time, the fasting sample was taken, registering the order of sampling for the different animals. Once these samples were taken, the animals were moved back to the grass field where they were fed *ad libitum* again. Although no quantification was performed on the amount of grass consumed, all the animals registered similar grassing activities and after 1 h they were all removed from the grass field back to the enclosure where they were sampled for post-feeding in the same order as in the first sampling. One animal, BON-C, bled during the post-feeding sampling, which contaminated the sample. Consequently, this sample was discarded.

The ruminal fluid samples of the BON animals were collected using a nasogastric probe, and the ruminal fluid of the Holstein animal was obtained directly from the rumen through a fistula according to the standard procedure published in Minor et al. (1977). The ruminal fluid samples were initially filtered through a sterile cheesecloth to remove plant material, then 200 mL of ruminal fluid was stored in 50 mL tubes with 10% (v/v) formal saline solution. The samples were refrigerated at 4°C until further use.

All animal procedures were conducted by a veterinary doctor after the previous approval of Agrosavia’s bioethics committee and followed all the guidelines and standards for animal care. In addition, the Institutional Committee for the Care and Use of Laboratory Animals (CICUAL) at the Universidad de los Andes also approved these procedures. The number of individuals used in this study was maintained to a minimum to avoid any negative impact of the research on animal health and wellness.

### Sucrose Density Gradient Construction

Eight solutions were prepared in a PBS buffer at different sucrose concentrations of 5, 10, 20, 30, 40, 50, 60, and 70% (w/v% or g/L). Each solution was prepared to a final volume of 100 mL. The sucrose gradient was assembled as follows. First, a 5 mL of a sucrose solution at 70% was added to a 50 mL tube, which was immediately immersed into liquid nitrogen for approximately 60–80 s, until the solution was completely frozen. Next, 5 mL of the 60% solution was added and fast-frozen as mentioned before. The process was repeated with the other sucrose solutions from the highest (50%) to the lowest (5%) sucrose concentration. The 50 mL tube was kept frozen at −20°C until further use.

From each of the three 50 mL samples collected for each animal for each treatment, a sucrose gradient was prepared. To prepare the sucrose density gradient for analysis, it was thawed for 2 h at room temperature, keeping it still to prevent the different sucrose solutions from mixing. A total volume of 5 mL of ruminal fluid was carefully added on top of the sucrose density gradient. The tube was centrifuged at 5,000 × *g* for 35 min at 4°C to prevent damage to the protozoan cells. After centrifugation, the fractions of each gradient were retrieved separately using a sterile 16 G needle, which was inserted through the wall of the falcon tube at the bottom end of each fraction. This process was performed sequentially starting from the top of the tube with the lowest concentration fraction (5%). The individual gradient fractions obtained from the previous procedure were stored in 15 mL tubes at 4°C until further use. The experimental replicates from each animal and treatment were pooled together in order to obtain DNA concentrations suitable for sequencing at specific sucrose gradient levels, this will hinder estimates of reproducibility as technical replicates were not assessed. In total, 69 samples were processed in this study.

### Microscopy Analysis of Sucrose Gradient Fractions

To evaluate the size distribution of the microorganisms in the different gradient fractions, an aliquot of 10 μL of each sample was examined under a light microscope. Some selected samples were used to visualize the microorganisms under the scanning electron microscope and confocal microscope. To reduce focal problems due to microbial cells accumulating at different depths, we used Colorfrost Plus Slide Glass, which electrostatically adhered the microbial cells to the slides (ThermoFisher, United States). All observations were performed at the Microscopy Center of the Universidad de los Andes. High-resolution pictures were taken with the scanning electron microscope to confirm the cell size observations. The size of the microorganisms in each fraction was measured using the confocal microscope and five independent photographs were analyzed using a custom ImageJ (Abramoff et al., 2003) script on each fraction from each sample (available upon request). The ImageJ script includes image preprocessing steps (background subtraction algorithms, noise filtering), segmentation (Phansalkar Local threshold method), and post-processing routines (watershed, hole filling, erode-dilate morphological filtering). We gaged the convex hull of each cell and computed its area value in square microns. We assessed image quality by using the deep neural network model proposed in Yang S. J. et al. (2018).

### DNA Extraction in Different Sucrose Gradient Fractions

DNA extraction of each gradient fraction was done as follows. First, 5 mL of each gradient fraction were centrifuged at 19,064 RCF at 1°C for 1 h. The supernatant was discarded and the pellet was washed with a PBS buffer to remove sucrose residues. The suspension was centrifuged again at 11,769 RCF at 4°C. The supernatant was discarded, and the pellet was resuspended in 200 μL of PBS buffer for a second time. The three experimental replicates for each gradient were pooled in a 2 mL centrifuge tube to make a single DNA extraction. In addition, 500 μL of a homogenized, composite ruminal fluid sample that did not undergo a separation by sucrose density gradient was also extracted and used as a total community for reference comparisons (bulk DNA extraction). The samples were frozen in liquid nitrogen for 5 min and were then placed in a water bath at 65°C for 5 min. This procedure was repeated three times in order to promote the lysis of the cells. DNA extraction was performed using the kit ZR Fungal/Bacterial DNA MiniPrep^TM^ from Zymo research following manufacturer’s instructions. Samples were initially homogenized using bead beating for 5 min. The DNA concentration was measured using the Nanodrop (ThermoFisher, United States) and the quality of the DNA was verified by electrophoresis in a 2% (w/v) agarose gel. The DNA extractions from the different fractions were quantified, obtaining concentrations ranging from 8 to 100 ng/μL (see Supplementary Table 1). All samples with concentration greater than 30 ng/μL were diluted to 30 ng/μL. For PCR (see below) 30 ng of DNA was used as template, implying 1 μL for all dilutions and the corresponding volume from the stock sample in order to reach 30 ng for samples with less than 30 ng/μL.

### Construction of Amplicon Libraries of the 16S rRNA Gene and Sequencing

The construction of metabarcoding libraries for *16S rRNA* gene was performed in two steps, following the protocol described by Caro-Quintero and Ochman (2015) which targeted the hypervariable region V4. In the first step, a triplicate PCR reaction was prepared for each of the samples. Amplification was performed using 30 ng of DNA template, 12 μL of the Promega GoTag^®^ Green Master Mix (United States), 10 μL of ddH_2_0, and 1 μL of each of the primers 515F and 806R at a concentration of 10 μM (Caporaso et al., 2012) with customized modifications to include a partial sequence of the Illumina amplification primers as shown in Faith et al. (2013); see Supplementary Table 2). The PCR amplification reactions were carried out under the following conditions: initial denaturation of 3 min at 94°C; three cycles of 45 s at 94°C, 30 s at 62°C, and 30 s at 72°C; three cycles of 45 s at 94°C, 30 s at 60°C, and 30 s at 72°C; three cycles of 45 s at 94°C, 30 s at 58°C, and 30 s at 72°C; 25 cycles of 45 s at 94°C, 30 s at 56°C, and 30 s at 72°C; and a final extension of 10 min at 72°C. PCR products were purified using AMPure XP beads (Beckman Coulter) following the manufacturer’s instructions.

In the second step, the triplicate amplification products were pooled together. Five microliters of this pool were used as a template for a second PCR reaction where the indexes and the Illumina adaptors were added to the sequences (Supplementary Table 2). The second PCR reaction was performed with the same conditions as described in the first PCR reaction. The PCR conditions included an initial denaturation of 3 min at 94°C, 12 cycles of denaturation for 45 s at 94°C, annealing for 1 min at 56°C, an extension for 1.5 min at 72°C, and a final extension of 10 min at 72°C. PCR products were cleaned using AMPure XP beads (Beckman Coulter). The quality of the DNA was verified by electrophoresis in a 2% (w/v) agarose gel. The DNA concentration was measured using Qubit 2 fluorometer (ThermoFisher, United States). An equimolar pool of all the PCR products was made with a final concentration of 10 nM. Pair-end libraries were sequenced in an Illumina MiSeq machine at the Universidad del Bosque in Bogotá, Colombia.

### Bioinformatic Analysis

Samples were demultiplexed with an in-house script (available upon request). The quality of the sequences was verified using FastQC v0.11.7 (Andrews, 2010). The adapters and linker sequences were removed from the *16S rRNA* gene partial sequences, using cutadapt v1.12 (Martin, 2011). Low-quality sequences, with an average Phred score <20 and having a read length <200 nts, were detected and removed from the datasets using trimmomatic v0.38 (Bolger et al., 2014). Qiime2 pipeline v_2018 (Bolyen et al., 2019) was used to analyze the *16S rRNA* gene amplicon libraries. Dada2 (Callahan et al., 2016) was the algorithm used to denoise and generate Amplicon Sequence Variants (ASVs). Pair-end sequences were used as input for Dada2 with a truncation length of 200 bp. The ASVs present in less than two samples or had less than 4 sequences in all the samples in the feature table were eliminated from further analyses. The taxonomic assignment of the ASVs was performed using the Silva Database version 132 (Glöckner et al., 2017).

### Microbial Community Structure and Diversity Estimates

All samples were rarefied to a depth of 7,200 sequences, which was the minimum number obtained for any given sample. We used Observed OTUs, Shannon and Faith’s PD indices as alpha diversity metrics to explore within-sample microbial heterogeneity. Beta diversity (between-sample diversity) was calculated using the weighted UniFrac metric (Lozupone et al., 2011). A principal coordinate analysis (PCoA) was used to visualize the results and the effect of the different variables under study such as the sucrose concentration, breed, individual, and sampling time. Rarefaction curves and beta diversity metrics were estimated using the “*core-metrics-phylogenetic*” plug-in implemented in the Qiime2 pipeline.

### Statistical Analysis

A permutational multivariate analysis of variance, PERMANOVA (Anderson, 2017), was performed using weighted UniFrac distance matrix on the ASVs table to establish if there were significant differences among groups of samples according to variables such as fractions of the gradient, the breed, and the individual animals. Distances were calculated among the different fractions of the sucrose gradient as well as the total sample of ruminal fluid. Pairwise comparisons (PERMANOVA between two groups) were made between gradient’s fractions and between animals to establish significant differences.

Welch’s test (White et al., 2009) was used to establish if there were significant differences in taxonomic families’ abundance or ASVs between fractions, and total, and between fasting and post-feeding treatments. In addition, the Fisher exact test (Ironyt and Pereirat, 1986) was used to quantify the enrichment of a given taxonomic group in the different fractions of the gradient. The taxonomic groups were filtered in both tests using a difference between proportions >1% together with an FDR adjusted *p*-value < 0.01. These two tests were implemented in the software STAMP-v2 (Statistical Analysis of taxonomic and functional Profiles) (Parks et al., 2014). To visualize if a taxonomic group (family or an ASV) was significantly enriched, an extended error bar plot was performed in the STAMP-v2 program.

An abundance matrix was generated with the taxonomic groups that had a statistically significant difference in at least two fractions of the gradient for the same animal. This matrix allowed us to identify in which fractions of the gradient a specific taxonomic group was enriched. The enrichment of the taxonomic groups across the gradient fractions was analyzed for each animal.

## Results

### The Sucrose Density Gradient Allows for the Separation of the Microbial Community by Size

We developed a methodology to disentangle the complex composition of the ruminal microbial community using sucrose-based fractionation of microbial cells. Our approach incorporated a sucrose step gradient using a range of eight sucrose (w/v) concentrations: 5, 10, 20, 30, 40, 50, 60, and 70%. We also included the densest fraction of the community that moved through all gradients and formed a large pellet at the bottom of the tube (labeled 70%_F) which was enriched with protozoa that were not observed in any of the other fractions. Ruminal samples were collected from four animals at *fasting* and *post-feeding* treatments, with the exception of one animal (BON-C) who was not possible to sample at 1 h post-feeding, for a total of seven ruminal samples. For each of those samples, the nine step gradient fractions were obtained as well as a total non-fragmented sample, resulting in 69 samples.

**FIGURE 1:**
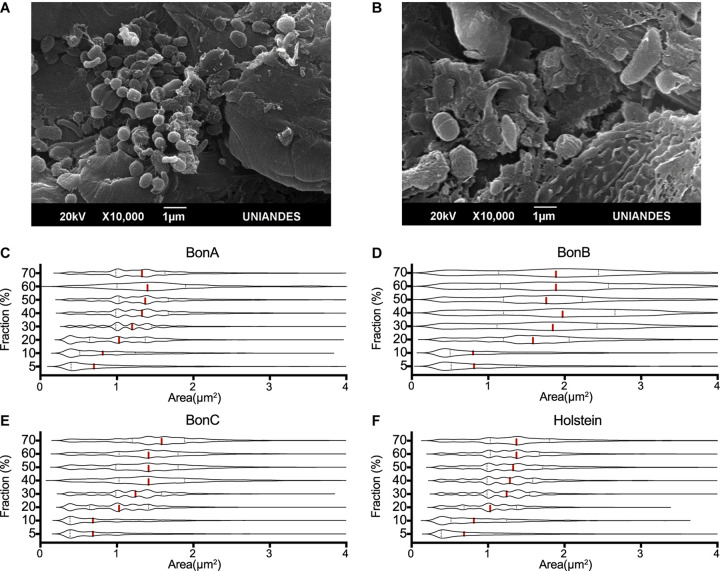
Cell size distribution of rumen microorganisms retained by the different sucrose fractions. The transmission electron microscope images show the differences in the size of microorganisms present in the **(A)** 20% and **(B)** 70% sucrose fractions. Violin plots represent the distribution of cells’ area quantified by confocal microscopy, five confocal microscopy microphotographs were analyzed for each gradient and animal. The panels show the distributions of the 70, 60, 50, 40, 30, 20, 10, and 5% fractions, for the animals **(C)** BON-A, **(D)** BON-B, **(E)** BON-C and **(F)** Holstein.

Using electron microscopy, it was observed that the lowest density fraction (5%) enriched the smallest bacteria, with an average size ranging from 0.2 to 0.8 μm^2^, while the larger microorganisms of about 1–4 μm^2^ in area were enriched in the larger fractions (30–60%) (Figure 1A). Other large microorganisms such as protozoa and bacterial clusters (i.e., chains and aggregates) were found in the highest density fractions (i.e., 70 and 70%_F) (Figure 1B).

To assess the consistent separation of similar size microorganisms, five confocal microscopy microphotographs were analyzed for each gradient and animal. The cell size (area) was determined for each microphotograph, and the area distribution was represented as violin plots. Although an overlapping distribution in cell sizes along the gradient was observed, different cell sizes were clearly enriched at different concentrations of the gradient. The cell size distribution for samples corresponding to the BON-A (Figure 1C), BON-B (Figure 1D), BON-C (Figure 1E), and Holstein (Figure 1F) showed that the step gradients demonstrated similar size selection across animals. For instance, the distributions of the 5 and 10% fractions showed an enrichment of small cell-sizes, with the first quartile and median of the cell size around 0.3 and 0.7 μm^2^, respectively. In the case of the 20% gradient, the first quartile was around 0.75 μm^2^ with a median around 1 μm^2^. For the 30% gradient, the first quartile was around 1 μm^2^ with a median around 1.3 μm^2^. Finally, for the concentrations between 40 and 70%, the first quartile was around 1 μm^2^ and the median was around 1.5 μm^2^. For BON-B, cell size distribution for all gradients seems to have a larger variability and wider distribution due to suboptimal focus quality. We obtained absolute measure predictions of image focus with no user-specified parameters, using the deep learning trained model (Yang S. J. et al., 2018), combining all images for BON-B and comparing the focus accuracy with that of the images from BON-A. A clear decrease in accuracy could be observed (Supplementary Figure 1).

### Diversity Is Mainly Driven by Sucrose-Based Fractionation

To analyze the microbial community structure in bovine ruminal fluid and the effect of the sucrose-based fractionation, we sequenced the V4 region of the *16S rRNA* gene. An average sequence depth of 33.661 ± 60.044 (mean ± SD) reads were obtained per sample. ASVs were identified using Dada2 and rarefaction curves for each sample were calculated for all samples (Supplementary Figure 2). Given the diversity saturation observed for most samples at ∼7,000 reads, a rarefaction was performed at 7,200 sequences to prevent sample loss and used for downstream analyses.

**FIGURE 2:**
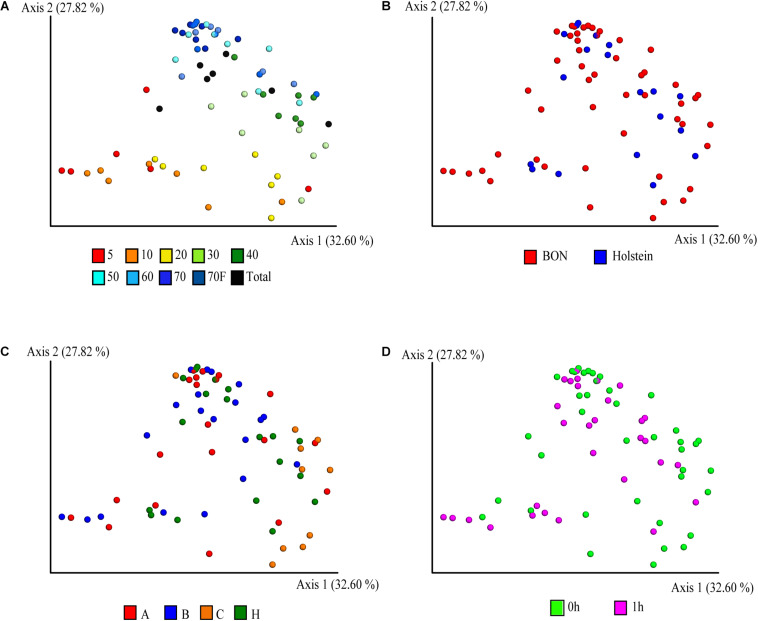
Principal coordinate analysis (PCoA) of the fractionation of the ruminal microbial community based-on weighted UniFrac distances. Distribution of the community in the samples is depicted according to **(A)** sucrose concentration, **(B)** breed, **(C)** animals, where A, B, C, and H represent BON-A, BON-B, BON-C, and Holstein respectively, and **(D)** sampling at fasting and post feeding.

The PCoA with weighted UniFrac distance metric on the ASVs abundances was used to visualize similarities between the bacterial communities and evaluate the effect of variables such as sucrose concentration, breed/sampling method, individual, and treatments. Interestingly, the sucrose concentration was the variable with the highest contribution at inter-sample diversity, as it corresponded to the variable that principally explained the variation in the first two axis of the PCoA with 32 and 28% of the variability explained, respectively (Figure 2A and Supplementary Figure 3). In general, a distinction was observed for the 5, 10, and 20% fractions compared to the rest of the fractions, suggesting that they share a similar bacterial composition, confirming what was also observed on the cell size distribution by the confocal microscopy (Figure 1). The total (non-fragmented) samples of ruminal fluid were grouped closely together with the largest gradient fractions (50, 60, 70, and 70%_F), suggesting that the total ruminal fragment is enriched with bacterial taxons more commonly identified in the medium-high density gradients.

**FIGURE 3:**
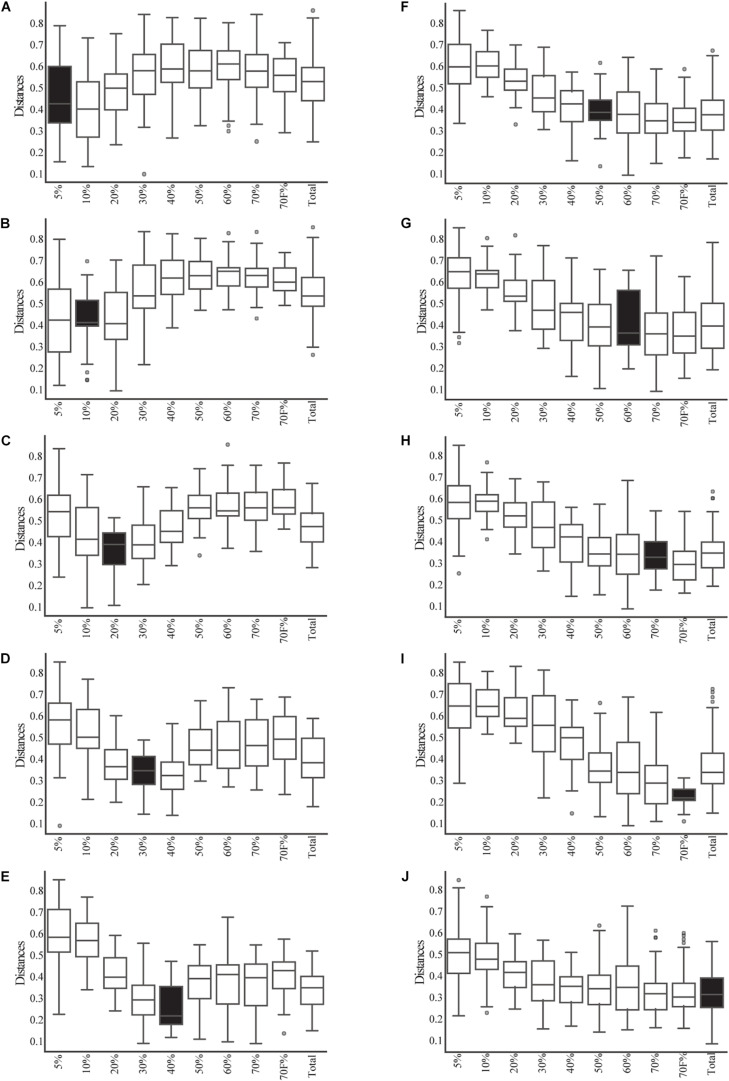
Beta-diversity analysis based on weighted UniFrac between and within the fractions of the gradient and total samples. The distribution of the distances between each sample from each fraction of the gradient (and the total) and the samples from each other fraction of the gradient are shown in open box plots. The distances of samples within a given fraction of the gradient are shown in black box plots. **(A–I)** Correspond to each fraction of the gradient. **(J)** Corresponds to the distance of the total samples compared to all gradients and itself.

Given that most of the variation was explained by the fractionation of density gradients, a PERMANOVA analysis was performed to test whether the weighted UniFrac distances among the different gradients were significantly different (pseudo-*F* = 5.39, *p*-value 0.001, 999 permutations; see Supplementary Table 3A for pairwise comparisons). This analysis showed that the smallest fractions of the gradient (5 and 10%) and the larger fractions (50, 60, 70, 70%_F, and Total) are more similar. This can also be observed when comparing weighted UniFrac distances within and between gradient fractions (Figure 3). For example, when comparing the distances within the different samples of the 5% fraction (Figure 3A, it is possible to observe black box) that they had lower variation. This low beta diversity or distance was also observed when comparing the samples of the 5% fraction with the 10 or 20% fractions. In contrast, significantly higher distances were observed when comparing the 5% fraction with the larger fractions (50, 60, and 70%). The behavior observed with the distances based on the composition of the microbial communities was similar to what was observed in the microscopy pictures. Importantly, some of the lowest inter-sample distances observed were between the total sample and each gradient, showing that the total is a composite of all the other samples.

Beyond the sucrose density gradient effect, a separation between breeds was observed in the PCoA plot on axis 3 (8.6% of total variation), where the samples belonging to animal of the Holstein breed were separated from those from the BON breed (Supplementary Figure 3B). It is important to mention that the Holstein animal was the only one sampled by cannula and not by nasogastric tube as the other BON animals. Several studies have suggested that there are no, or very minor, variations in the microbial communities obtained depending on the sampling method of rumen microorganisms (Kittelmann et al., 2015; Tapio et al., 2016; de Assis Lage et al., 2020). However, due to this complete co-variation between the sample treatment and the breed of the animals, it is impossible for us to determine if the breed was the main factor affecting the observed variation and not the sampling method. The samples belonging to the three BON animals were similar among them and no clear separation was observed (Figure 2C and Supplementary Figure 3C). Those results were further confirmed with the ANOVA analysis, where we found significant differences driven by breed/sampling method (pseudo-*F* = 3, 11,131, *p*-value = 0.003, 999 permutations) and individuals (pseudo-*F* = 4.154, *p*-value = 0.001, permutations = 999), although no significant differences were found between the individuals of BON-A and BON-B (Supplementary Table 3B). Finally, no apparent effect of the feeding was observed on the first 3 PCoA axes (Figure 2D and Supplementary Figure 3D).

### Enrichment of Bacterial Families and ASVs Throughout the Sucrose Density Gradient

**FIGURE 4:**
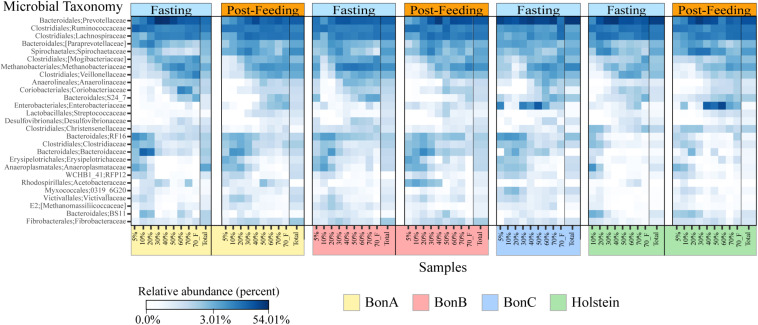
Heatmaps depicting relative abundance of the bacterial families across the sucrose density gradient fractions and the total sample for the BON-A **(A)**, BON-B **(B)**, BON-C **(C)**, and Holstein **(D)** at the time of fasting and post feeding. Note that no post-feeding sample was obtained for the BON-C. Relative abundance (percentage) is shown in a log scale to help visualize changes in the community composition of families that are not highly abundant. Families present in the four animals are highlighted in red. A black line divides the Total sample from each of the fractions.

Bacterial taxonomic groups were enriched in specific sucrose fractions compared to the total ruminal fluid. Figure 4 shows bacterial abundance at the family level for all the individual animals during fasting and post-feeding, for the families comprising up to 70% of the relative abundance. Several interesting observations can be derived from Figure 4. First, the families present in any gradient were also found in the total samples; additionally, all 27 bacterial families were present in all four animals. Second, as noted above, the 5 and 10% fractions have very similar composition, and for most of the animals this similarity extended to the 20% or even 30% fraction. However, the abundance of the families that were enriched in the 5 and 10% fractions began to decrease as the sucrose concentration increased. Examples of the families enriched in the 5 and 10% fractions included members from the Bacteroidales order, such as Bacteroidaceae and RF16, the Clostridiaceae and Erysipelotrichaceae from the Firmicutes, and the Anaeroplasmataceae from the Tenericutes. There were few families enriched in the 40 and 50% fractions, with the Enterobacteriaceae among those, suggesting that these fractions are a transition between smaller and larger bacteria. Additionally, other families were enriched only in 60, 70, and 70%_F, such as the families Mogibacteriaceae, Veillonellaceae, Methanobacteriaceae, Anaerolinaceae and S24-7 from Bacteroidales. Lastly, the 70%_F fraction shows different abundance patterns with respect to other fractions immediately preceding it, likely due to the presence of sessile bacteria that were attached to large particles and the absence of planktonic cells, prevalent in the other fractions. Families such as Prevotellaceae, Ruminococcaceae, Lachnospiraceae, and Spirochaetaceae were present in all the gradient fractions and were the most abundant bacterial families for all the animals.

Looking deeper in the taxonomic level, the distribution of genera per gradient shows three different patterns based on their relative abundance (Supplementary Figure 4), confirming the results observed at the family level. First, some genera were enriched at the lower sucrose concentrations (5, 10, and 20%), such as *Anaeroplasma* (*Anaeroplamataceae* family), RFN20 (*Erysipelotrichaceae*), *Clostridium* (*Clostridiaceae family*), BF311 (*Bacteroidaceae* family); all those genera belong to families identified as enriched in the smaller gradients. Furthermore, the genus *Anaerostipes* from the Lachnospiraceae family also was identified in these fractions. Second, we observed microorganisms with a higher relative abundance at more dense gradient concentrations (50, 60, and 70%); among these we found genera with species that have very large cell sizes such as *Selenomonas* (Veillonellaceae), *Oscillospira* and *Ruminococcus* (Ruminococcaceae), and *Butyrivibrio* (Lachnospiraceae). Finally, some genera have a higher relative abundance at the intermediate gradients (30, 40, and 50%); among these we found *Prevotella*, *Fibrobacter*, *Pseudobutirivibrio*, *Treponema*, and *Coprococcus*. Interestingly, when analyzed at the genus level, families such as Lachnospiraceae and Ruminococcaceae that were reported at high abundance in all fractions of the gradient were composed by different genera, each one with specific distribution of cell sizes highlighting the large diversity within these taxonomic groups.

### Bacterial Community Changes Are Better Detected Using the Sucrose-Based Fractionation

In order to evaluate the usefulness of the fractionation on the identification of subtle changes in the composition of the microbial community, we identified the taxonomic groups that show significant differences when comparing the fasting and post-feeding treatment for the same animal. It is important to highlight that the BON-C is excluded from these analyses due to the lack of post-feeding samples. We found significant differences in the abundance of bacterial families before and after feeding; many of those differences were not detected in the total samples (Supplementary Table 4). Using the program STAMP, we evaluated the differential abundance between fasting and post-feeding conditions at the family level (Fisher’s exact test, FDR corrected *q*-value < 1e−2) for each of the animals. We chose families that had an effect size (differences between proportions) greater than 1 (significantly enriched in fasting) or less than −1 (significantly enriched in post-feeding), comparing equivalent fractions (Supplementary Table 4). In general, it was observed that all the enriched families were identified either uniquely in the fractions or shared by the fractions and the total. Furthermore, the magnitude of the effect size and the percent of families enriched were greater in the fractions compared to the total sample. Specifically in terms of percentage of enriched families in fragments vs. totals, we have in BON-A: 81.5 vs. 37.0%; BON-B: 77.8 vs. 7.4%; Holstein: 66.7 vs. 22.2%. These results indicate that the gradients allowed the detection of a stronger family enrichment signal.

The families enriched exclusively in the fractions of the gradient included Coriobacteriaceae, Bacteroidales RF16 and Methanomassiliicoccaceae. On the other hand, the families that were present in all fractions showing consistent enrichment patterns included Ruminococcaceae, Lachnospiraceae, Spirochaetaceae, and Prevotellaceae. Interestingly, the enrichment direction was not the same on all animals or between the fractions and the total. An example of this is Prevotellaceae, which shows opposite treatment enrichment. In BON-B, this family is enriched in post-feeding in most of the gradients, while in BON-A it is highly enriched in the 20–50% gradients during fasting and in the total during post-feeding. Given that within a bacterial family a large functional and phenotypic diversity of microorganisms is expected, we examined the enrichment of microorganisms using ASV as a proxy for species level. We wanted to determine if at a finer level of taxonomic classification implying closer evolutionary relationships, a defined group may have a more restricted associated cell size, and therefore it would be possible to see stronger effects of separation on the enrichment at this level.

Using the total number of ASVs, we calculated the difference in proportions between fasting and post-feeding, analogous to what was performed at the family level, selecting those with enrichment in fasting (as a difference greater than 1) or post-feeding (with values less than −1). We selected only those ASVs that were significantly enriched in at least two fractions of the gradient (Supplementary Table 5). Analogously to what was observed at the family level, there was a higher number of ASVs enriched in the gradients compared to the number enriched in total samples. In summary, the proportion of enriched ASVs in fractions vs. the total samples is 29 vs. 7 for BON-A, 18 vs. 2 for BON-B; and 21 vs. 3 for Holstein. The ASVs that belong to the taxonomic groups Bacteroidaceae BF311, Bacteroidales RF16, Ruminococcaceae, Clostridiales, and Tenericutes ML615J-28 were enriched in the smallest fractions (5, 10, and 20%) for the BON-A and BON-B. Meanwhile, ASVs belonging to *Prevotella* were mostly enriched in the middle fractions (30, 40, and 50%) for all the animals, as well as ASVs from Enterobacteriaceae in the Holstein. The ASVs that belong to *Methanobrevibacter* genus were enriched in the largest fractions (60, 70, and 70%_F) of the gradient for the BON-B and Holstein, and the ASVs belonging to the bacterial group WPS-2 were enriched in the biggest fractions of the gradient in the Holstein. Supplementary Figure 5 shows the unique and shared ASVs enriched in the three animals. Only four ASVs were shared by all three animals, two of them belonging to the *Prevotella* genus, and the other ones to the taxonomic groups Clostridiales and Acholeoplasmataceae. The larger number of enriched ASVs was found uniquely in Holstein and BON-A, with 13 and 12 ASVs, respectively. Another group of enriched ASVs is those present in BON-A and BON-B, 12 ASVs, while no ASVs were found shared between Holstein and BON-B. This shows that each animal has their unique ASV profile, with animals from the same breed sharing higher numbers of bacteria.

**FIGURE 5:**
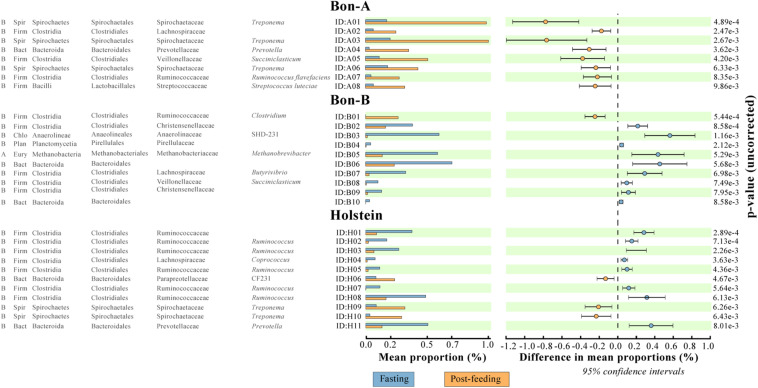
ASVs that were significantly enriched in all the fractions of the gradient before or after feeding. **(A)** BON-A, **(B)** BON-B, and **(C)** Holstein. The graphics were made using the program STAMP program using Welch’s test with a *p*-value < 0.01. No FDR correction was made.

**FIGURE 6:**
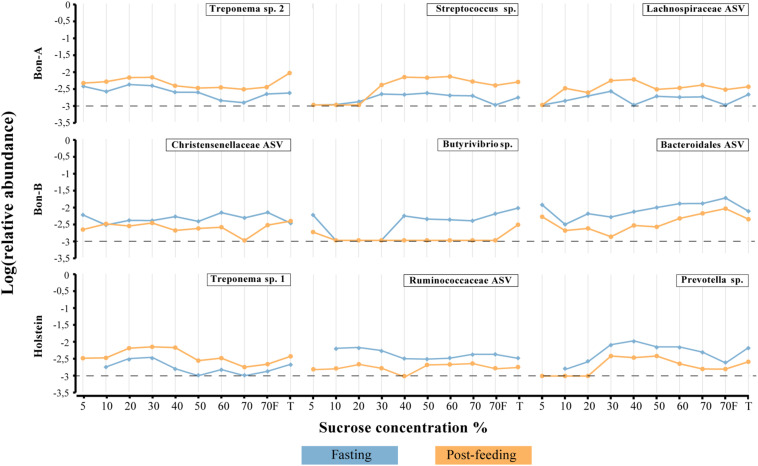
Relative abundance of the ASVs that show their enrichment throughout all, or almost all, the fractions of the gradient. **(A)** BON-A, **(B)** BON-B, and **(C)** Holstein. Blue lines correspond to the relative abundance while fasting and orange are the abundances 1 h post-feeding. Dotted black line at −3 represents a relative abundance of 1:1,000 which marks or limit of detection for the sequencing. The relative abundance was log-transformed.

**FIGURE 7:**
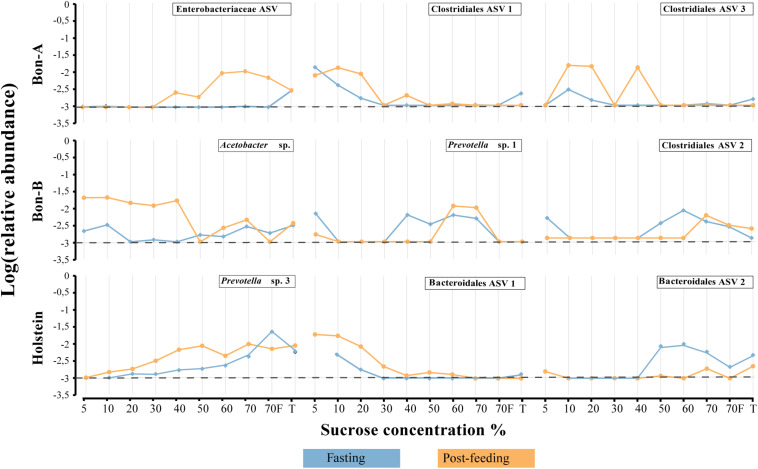
Relative abundance of the ASVs that show their enrichment in specific fractions of the gradient when the animals were fasting or post feeding. **(A)** BON-A, **(B)** BON-B, and **(C)** Holstein. Blue lines correspond to the relative abundance while fasting and orange are the abundances 1 h post-feeding. Dotted black line at −3 represents a relative abundance of 1:1,000 which marks or limit of detection for the sequencing. The relative abundance was log-transformed.

As observed at the family level, there were also some ASVs that were present in all the fractions of the gradients studied and in the total samples. It was possible to identify those ASVs enriched using a Welch test due to the treatment in most of the fractions (Figure 5), and for some selected representatives we plotted their abundance as a function of the sucrose fraction (Figure 6). The enriched ASVs are members of the genera *Prevotella*, *Butyrivibrio*, *Ruminococcus*, *Succiniclasticum*, *Paraprevotella*, and *Treponema*. Interestingly, some ASVs were enriched in between treatments but only in a few specific fractions of the gradient (Figure 7). Some ASVs were enriched in the smallest fractions of the gradient such as *Acetobacter* variants in the animal BON-B, other ASVs were enriched in the middle gradient fractions such as *Prevotella variants* in the animal BON-A, and other ASVs were enriched in the largest gradient fractions such as Enterobacteriaceae variants in the animal BON-A. The identification of those ASVs shows that the fractionation of the microbial community can detect changes in the abundance that total DNA extraction without fractionation cannot.

## Discussion

### The Sucrose Density Gradient and the Separation of the Ruminal Microorganisms

The ruminal microbial diversity is a key factor in improving productivity, health, and reducing methane emission in cattle (Matthews et al., 2018). Despite its importance, there are still gaps in the knowledge of the diversity of ruminal microbiota and we are still exploring how treatments and conditions affect the abundance and composition of ruminal microbes (Malmuthuge and Guan, 2017). In this study, we developed an alternative approach to study the composition and diversity of the ruminal microbiota by fractionating the microbial community based on cell size and density.

We were able to show that the sucrose gradient is a simple method for microbial community analysis, which showed consistent fractionation in four different animals tested. These results were confirmed both qualitatively and quantitatively by the cell size distribution of samples measured by confocal microscopy. Furthermore, it was shown that most cells are maintained intact in the different fractions, allowing the recovery of enriched cells viable for culture and other downstream analysis. A similar use has been reported in the human gut, where bacteria has been separated from the rest of the fecal material using a NICODENZ density gradient and could be recovered alive and preserved for a fecal microbiota transplantation (Hevia et al., 2015). We observed that the cell morphology and size distribution corresponded to that of bacteria, archaea, and protozoa; however, we did not observe fungi zoospores, an important part of this microbial community. A potential explanation is that zoospores attach to the plant material in a sessile state (Orpin and Bountiff, 1978). This matches with the observation that a very low number of motile zoospores have been observed in the ruminal fluid (Lee et al., 2000) and with our observation from the images obtained with the scanning electron microscope for the 40% fraction.

The analysis of cell size distribution clearly shows that even though breed/sampling method has an important effect on the ruminal microbial composition, same sucrose percentage fractions between different animals recover similar microbial taxonomic composition. In this sense, the cell size distribution obtained by microscopy and the taxonomic composition analysis showed a similarity between the 5, 10, and 20% fractions, significantly different from the 40, 50, 60, and 70% fractions. One possible explanation is that in the environment, cell sizes and their densities may differ to what has been characterized in laboratory conditions. They may depend on factors such as age, growth phase, or cell arrangement morphology (e.g., aggregates or chains); in consequence, the cell density might vary, as well as their distribution in the different gradient fractions. Because of this, it is expected to find ASVs in different fractions of the gradient. The lack of a finer resolution or separation among the fractions could be due to technical difficulties in recovering the purified fraction and/or incomplete fractionation during the centrifugation. A strong separation of the fractions by centrifugation was avoided to maintain the viability and integrity of the cells. Other plausible explanations of that seemingly bimodal distribution of bacterial sizes could be due to factors like protozoa predation and the anoxic environment. This variation in bacterial size has been reported as a consequence of predation of bacteria by the protozoa (Chrzanowski and Šimek, 1990; Gonzalez et al., 1990). Studies in aquatic environments have demonstrated that when bacteria are under grazing pressure, the bacterial size tends to become smaller or very large (Hahn and Höfle, 2001) as a size-defensive mechanism because protozoans prefer to feed on intermediate-sized bacteria (1 μm). Bacteria with a size smaller than 1 μm or greater than 2 μm have a predation rate 10–200 times lower than in an intermediate size bacterium. Another factor that could influence the similarity in the microbial composition in the fractions of 40–70% is the possibility of the protozoa disrupting the gradient as they pass through, generating a homogenization of the fractions.

### The Enrichment of Families and ASVs in the Sucrose Density Gradient

During the development of this research, it was possible to observe that the most important factor affecting the taxonomic composition of the samples was the sucrose gradient. This observation shows the possibility to use such methods for enrichment of certain bacterial species, genera, or families based on their size or density. At higher taxonomic ranks (i.e., family), it was possible to observe a consistency in the enrichment among the different gradients between animals, even from different breeds. For example, the taxonomic groups Anaeroplasmataceae, Bacteroidaceae, and Clostridiaceae were enriched in the smallest fractions of the gradient, which was consistent with what was observed at the genus level, with genera such as *Clostridium* (Ruminococcaceae); *Anaerostipes* (Lachnospiraceae); *Clostridium* (Clostridiaceae); BF311 (Bacteroidaceae); *Anaeroplasma* (Anaeroplasmataceae); RFN20 (Erysipelotrichaceae). Most of these genera have a reported small cell size, for instance *Anaeroplasma* and anaerobic mycoplasmas have an average cell size of 0.5 μm and *Anaerostipes* has a described cell size of 0.5–0.6 μm wide × 2.0–4.3 μm long.

Families enriched in the larger fractions of the gradients included Mogibacteriaceae, Veillonellaceae, Anaerolinaceae, Coriobacteriaceae, and S24-7. These families were further confirmed at the genus level, with members of the *Selenomonas*, *Anaerovibrio* (Veillonellaceae); *Ruminococcus*, *Oscillospira* (Ruminococcaceae); *Butyrivibrio* (Lachnospiraceae); *Methanobrevibacter* (Methanobrevibacteriaceae); SHD-231 (Anaerolinales); and *Desulfovibrio* (Desulfovibrionales). This includes organisms known for a large cell size such as the genus *Selenomonas* with an average cell size of 2.0–3.0 × 5.0–10.0 μm (Falkow et al., 2006) and Oscillospira has an average size of 3–6 × 10–40 μm (Gophna et al., 2017). Other genera, even though they have smaller reported cell size, are known to grow in filamentous and chain structures, such as *Butyrivibrio* (0.21–0.32 × 2–4 μm) (Sewell et al., 1988) and *Ruminococcus* (0.9 × 1.3 μm) (Latham et al., 1978). Another example of how the cell size influences the distribution across the fractions of the gradient of the bacterial group at the genus level is the *Ruminococcus* genus. The *Ruminococcus* are prevalent in most of the ruminant species, and they are major cellulose and cellobiose degraders (Krause et al., 1999). The bacterial morphology of *R. albus* is mainly coccus and diplococcus with diameters varying from 0.8 to 2.0 μm. The cells of *R. flavefaciens* are spherical with a diameter varying from 0.7 to 1.6 μm; however, this species grows in chains (Puniya et al., 2015), which is likely the reason why they were enriched in the 60 and 70% fractions. There were also families that appeared throughout the whole range of fractions such as Ruminococcaceae, Lachnospiraceae, Prevotellaceae, and families belonging to Clostridiales. Interestingly, at the genus level, we observe genera from these families being enriched in different fractions. These families, which are among the most abundant and prevalent in the ruminal microbiota (Henderson et al., 2015), have been described with large morphological diversity living as single cells, filaments, chains, and flocs (Parte et al., 2011).

One important aspect is the enrichment of unclassified or poorly characterized bacteria in different gradients. We observed ASVs from the candidate division SR1 (SR1c) and Tenericutes phylum were only enriched in the small fractions of the gradient. The SR1c group is also found in diverse environments like sea sediments (Corre, 2001), termite gut (Hongoh et al., 2003), and sulfur rich springs (Blank et al., 2002) and do not have any representative isolated strains. The SR1c has been reported in several of the rumen microbiota studies (Davis et al., 2009; Zhong et al., 2018). However, the diversity of this group in the rumen is low, and only two OTUs from this group have been reported in the rumen and belonging to the BD2–14 sub-group III (Davis et al., 2009; Ghotra, 2014; AlZahal et al., 2017). The SR1c was also present in all the fractions of the gradient indicating that there are other bacterial sizes of this group in the ruminal fluid.

### The Sucrose Density Gradient as a Tool to Disentangle Subtle Changes in Microbial Composition in Response to Treatments

In our experimental design, we aimed at using the fractionation of cells according to their density to allow for a higher resolution of changes and enrichments upon treatments such as feeding. Although the treatment used (fasting vs. feeding) does not necessarily imply a drastic change in the microbial community, most of the variations and enrichments observed were found precisely in the fractions and not in the total samples. In this experiment, the post-feeding time could actually represent an active phase of transitions with a reported peak of increased abundance of fibrolytic bacteria at 1–2 h post feeding (Huws et al., 2018). Examples of these increases in abundance were the families Bacteroidales BS11 and Bacteroidales S24-7. These families are commonly present in the rumen but are found in low abundance and do not have cultured representatives yet. In our study, the family BS11 was enriched in the 10 and 20% fractions, for the BON-A when fasting, in the 5 and 10% for the Holstein, and in the 50% fraction in the BON-B post-feeding (Figure 4), suggesting a niche partitioning of this family in the rumen (Solden et al., 2017). The presence of this family in different fractions could indicate different genera with different size and functional profiles in the rumen. The family Bacteroidetes S24-7 was enriched in 60, 70, and 70%_F for most of the animals when they were fasting (Figure 4), which could be explained by the bacterial cells floating in the ruminal fluid waiting to adhere to the plant material that enters the rumen when the animal feeds on grass. To obtain more precise results about the enrichment of the different taxonomic groups when fasting or post feeding, it would be desirable to sample a larger number of animals to observe more robust and homogeneous trends. On the other hand, the functional response of the low abundant microorganism, such as the families Bacteroidales BS11 and S24-7, is not well understood in ruminal animals. The enrichment of these families in the gradient will provide an opportunity to explore the genome of these low abundant groups. In this sense, recent studies performed in the human gut microbiome demonstrated the importance of the low abundant microorganisms as a source of new functional genes with antimicrobial and biotechnological applications (Almeida et al., 2019).

We believe that the current method is widely applicable and of potential interest in different relevant situations, particularly when studying the effect of treatments such as prebiotics and probiotics in the animal microbial community since some of those effects are more subtle and may not affect the most abundant bacteria. However, even small changes may bring significant biological variation. The method presented here can be further optimized, for instance, the addition of a cell dissociating step prior to the fractionation in order to separate those microorganisms that can flock together, or those that are attached to large plant material. The dissociation can be performed using treatment such us Tween or physical methods such as sonication. Based on our results is not clear whether the complete set of different gradients is needed in order to obtain the observed separation, as each gradient sieves the bacteria that pass to the next gradient, or if a selected subset of gradients could have the same effect. However, from the obtained results it is clear that is not needed to sequence all fractions. We suggest that in future studies a focus could be made on the 5, 30, 60, and 70_F fractions. We think this will reduce the sequencing cost, while still representing all of the taxonomic groups that inhabit in the rumen. As with any enrichment method, it is important to consider that any bias or noise that could be present in the samples could be artificially enriched when separated throughout the sucrose gradient.

For other microorganisms, an optimization of the gradient or even the solution will still be necessary. In the current setup, the viral component of the rumen community will be present only in the 5% gradient fraction. If what is desired is to fractionate the different members of the viral community, a Cesium Chloride gradient with ultracentrifugation should be used instead. On the other hand, when considering large organisms such as protozoa, the sucrose density gradient facilitates the isolation and concentration of these microorganisms in a unique fraction making it easy to determine the effect of a treatment on their abundance.

Finally, caution is important when analyzing the enrichment based on specific fractions of the gradient. If the total DNA yield of a given fraction is too low or the alpha diversity is significantly affected compared to the total sample, it is possible that sampling bias could affect the relative abundance of the taxons and thus the enrichment observed. Furthermore, if biological inferences will be made based on enrichments identified in particular fractions, verification through qPCR or other methods such as CFU counts on selective media should be done, starting from the total sample, to confirm the results obtained from the fractions.

In conclusion, in this study we describe a low-cost methodology to expand the knowledge of the ruminal microbial diversity in the ruminal fluid compared to a standard procedure. Using the sucrose density gradient methodology, we separated the ruminal microbiota based on their size and density in different fractions of a gradient. We demonstrated that fractions of the gradient of ruminal sample fluid have different compositions and abundance, which allowed us to enhance the resolution of the study of bacterial diversity in the rumen. We found an enrichment of the less abundant bacterial families and ASVs in different fractions of the gradient compared with a total sample without separation. Lastly, the sucrose density gradient was able to detect subtle changes in the differential abundance, not only for the most abundant and prevalent groups of bacteria in the rumen before and after feeding, but also for the less abundant groups. These less abundant groups are likely equally important groups in the rumen. Hence, this method will allow an improved diversity analysis of the effect of a treatment on the ruminal microbiota. The enrichment of specific or less abundant taxonomic groups in specific fractions of the gradient maintaining their viability in a less complex mixture open the possibilities for culturing strategies or even whole-genome shotgun sequencing for genomic reconstruction, paving the way for a more exhaustive functional characterization of those elusive members of the ruminal microbiome.

## Data Availability Statement

The datasets presented in this study can be found in online repositories. The names of the repository/repositories and accession number(s) can be found below: https://www.ebi.ac.uk/ena, PRJEB42578.

## Ethics Statement

The animal study was reviewed and approved by Committee for the Care and Use of Laboratory Animals (CICUAL), Universidad de los Andes.

## Author Contributions

HJ, AC-Q, RH, and AR contributed to the conception, design, and supervision of this study. HJ and AC-Q provided resources for the field sampling and the processing of the samples in the laboratory. AR supplied the computational resources for the bioinformatic analysis. Data collections were made by RH and HJ and the processing and analysis of the data was made principally by RH with collaboration from AR and AC-Q. CV-G processed and analyzed the data of the images obtained with confocal microscopy. The statistical analysis was made by RH and AR. RH wrote the first draft of the manuscript. HJ, AC-Q, CV-G, and AR participated in the writing of different sections of the manuscript. All authors read, contributed to manuscript revision, and approved the submitted version.

## Conflict of Interest

The authors declare that the research was conducted in the absence of any commercial or financial relationships that could be construed as a potential conflict of interest.
