# Supplementary material for: Disentangling the Complexity of the Rumen Microbial Diversity Through Fractionation Using a Sucrose Density Gradient
Source: Front Microbiol. 2021 Jul 8;12:664754. doi: 10.3389/fmicb.2021.664754 (PMC8297521; doi:10.3389/fmicb.2021.664754)
Supplement: Supplementary file 1 [file Data_Sheet_1.pdf]

Supplementary Figures

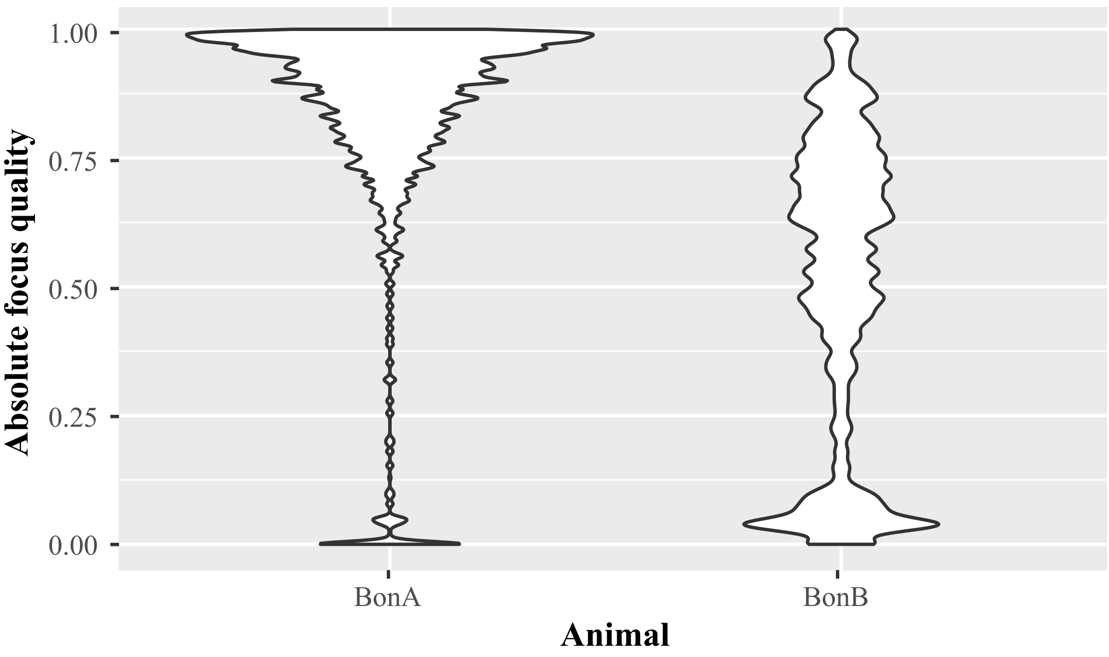

Figure S1. Focus quality of microscopy images. We compute an absolute focus quality score through the deep learning based tool proposed in (Yang 2018). The deep learning model was trained using 300 plus in-focus nuclei stain images of U2OS cells, which were synthetically defocused to one of 11 absolute defocus levels during training. The violin plot shows in the y axis the probability of a pixel being in an image region that is at the best quality focus level. Scores are shown for two animals, BonA and BonB.

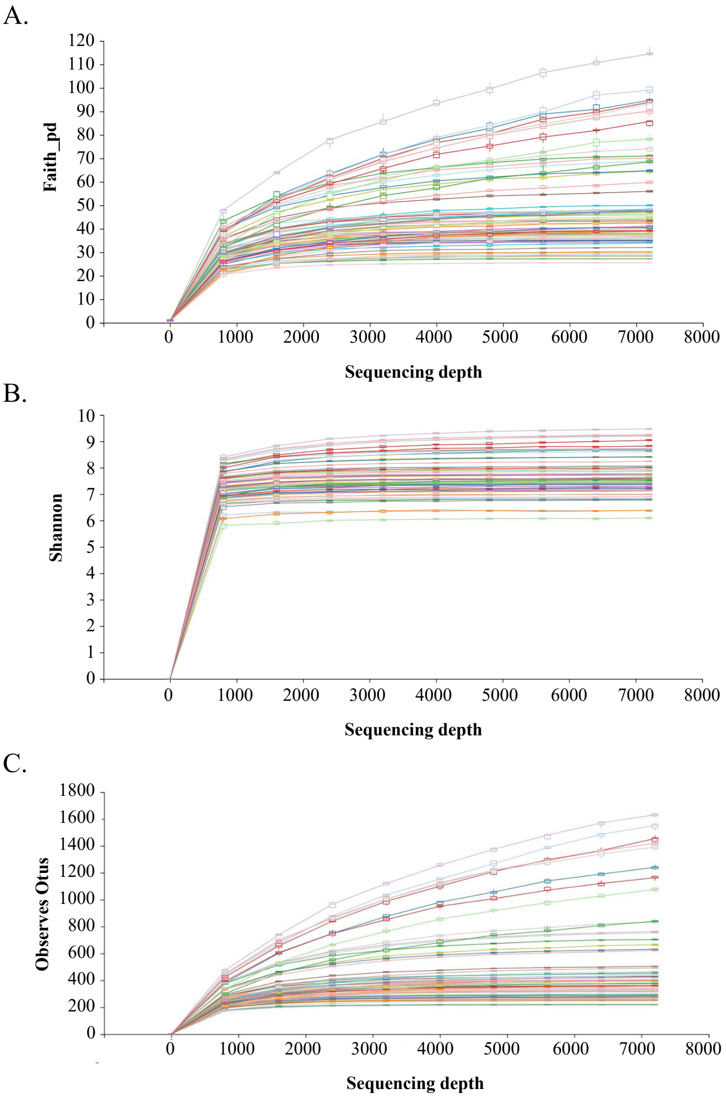

Figure S2. Rarefaction curves for each of the fractions of the gradient and the total samples of ruminal fluid in all the animals for three different metrics: Faith-PD (A), Shannon diversity index (B) and Observed OTUs (C).

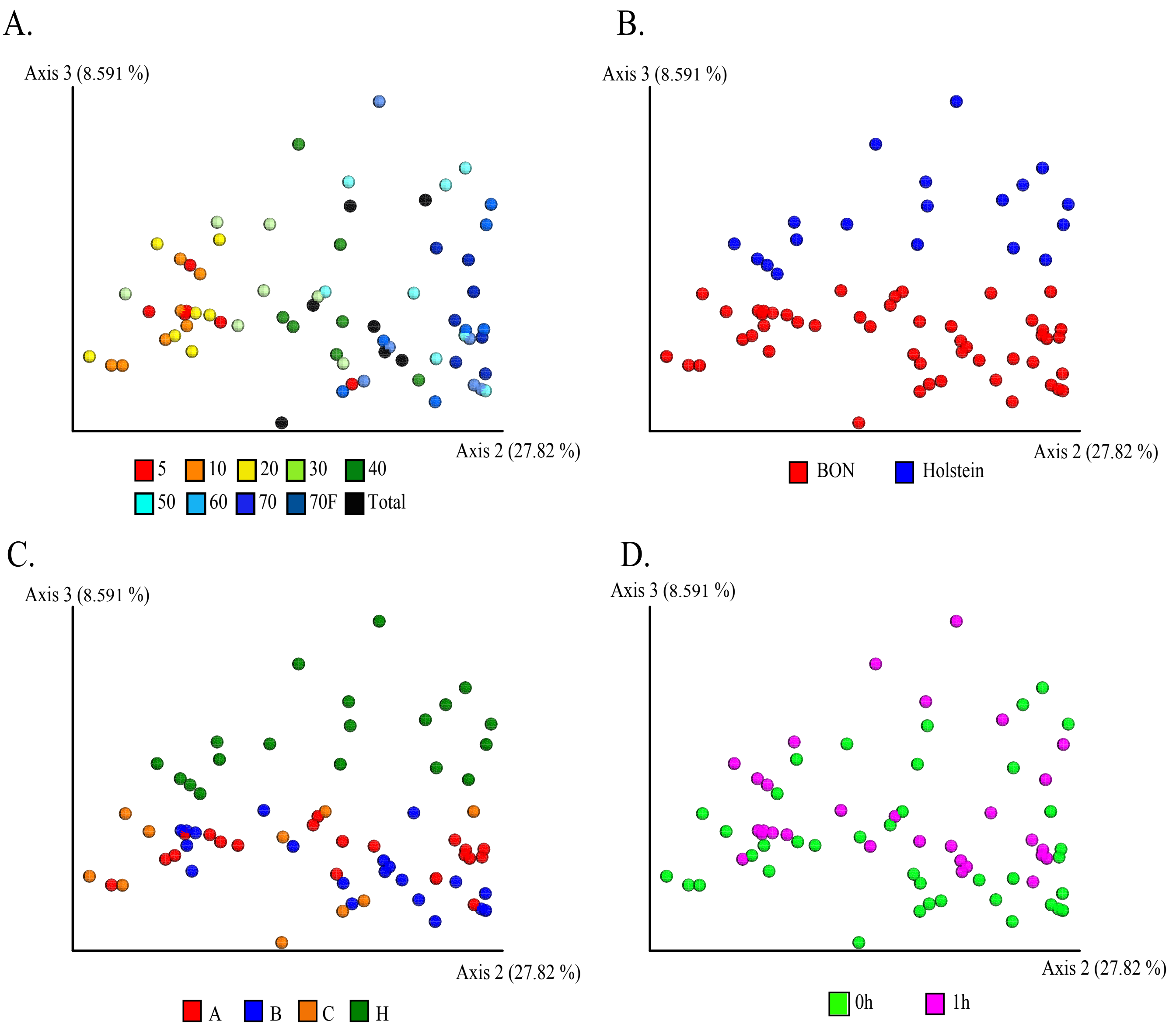

Figure S3. Principal Coordinate Analysis (PCoA), second and third principal coordinates of the fractionation of the ruminal microbial community based on weighted UniFrac distances. Distribution of the community in the samples are depicted according to: (A) Sucrose concentration, (B) Breed/sampling method, (C) Animals and (D) Time of sampling (before or after feeding).

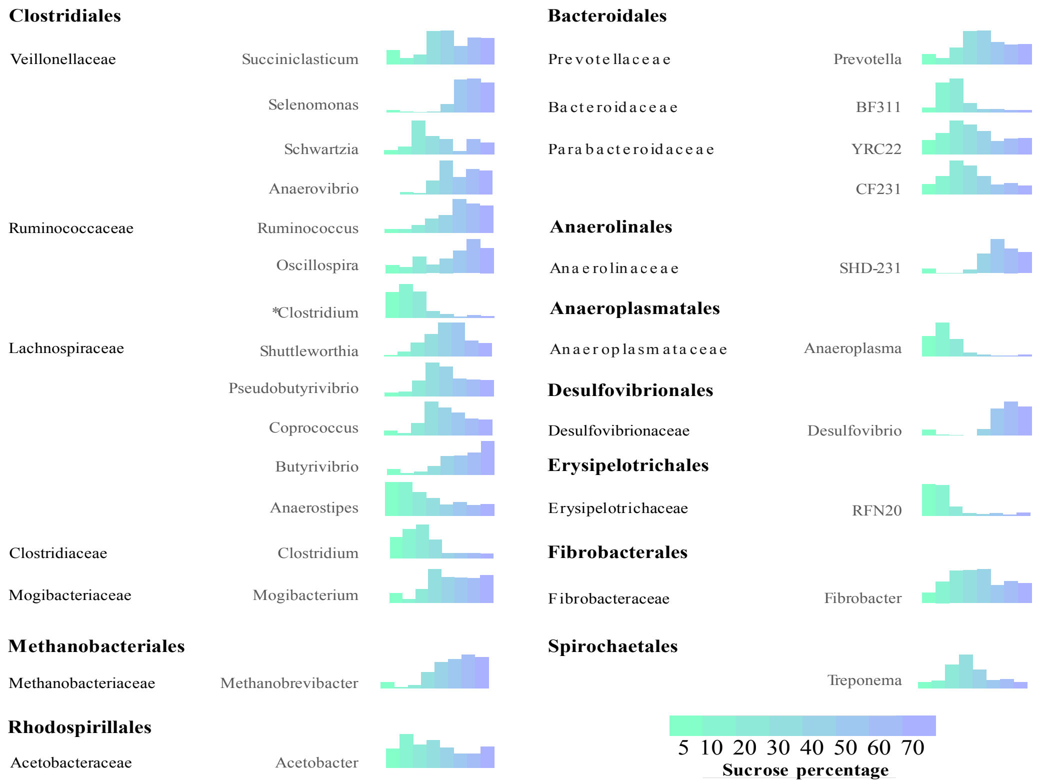

Figure S4. Genus relative abundance distribution per gradient.The cell size distribution was determined for genera with higher than 0.1 % of relative abundance. For each genus, the values of relative abundance per sucrose gradient were determined by adding the relative abundance of their corresponding ASVs. The addition was done for all animals and treatments, we assumed that even though host or treatment change the relative abundance of ASVs, they do not have a big effect on bacterial size distribution.

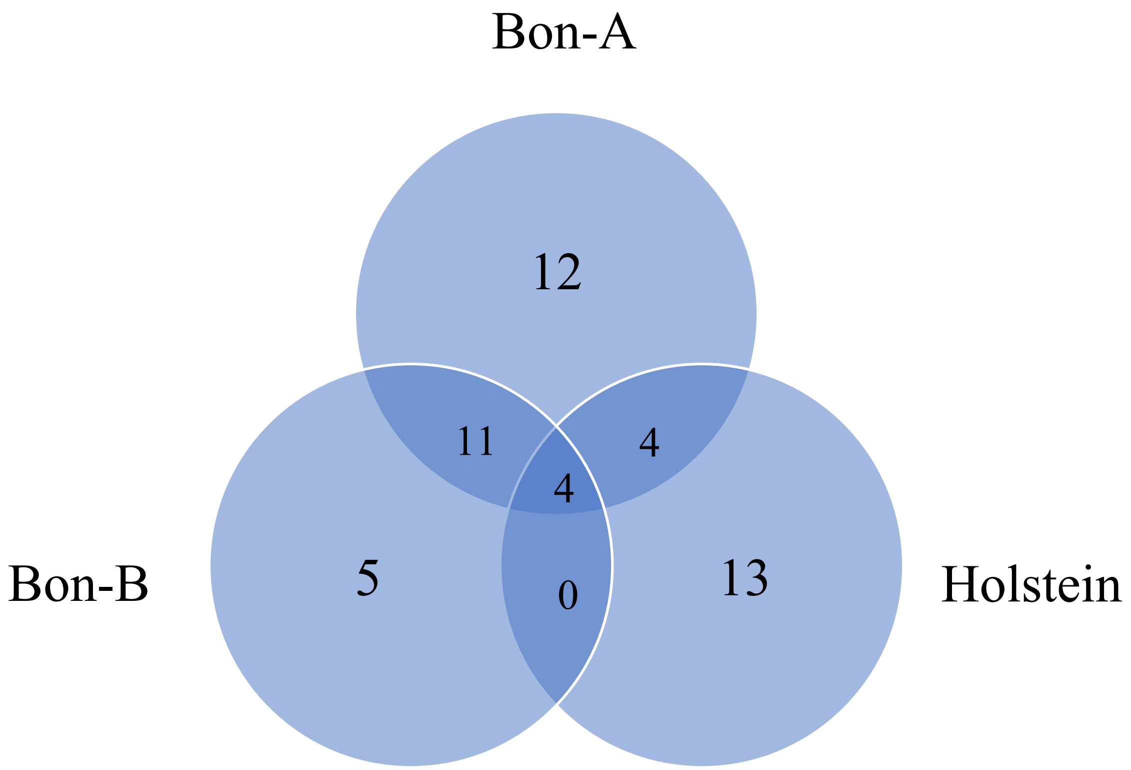

Figure S5. Venn diagram showing the number of ASVs that were shared between the animals BON-A, BON-B and Holstein
